# Fostering cross-community collaboration to advance pandemic and epidemic intelligence

**DOI:** 10.1186/s12919-026-00399-9

**Published:** 2026-09-21

**Authors:** Julia Fitzner, Barbara Tornimbene, Hugo Gruson, Adam Kucharski, Kevin J. Olival, Chloe Rice, Lisa Waddell

**Affiliations:** 1 World Health Organization (WHO), WHO Hub for Pandemic and Epidemic Intelligence, Berlin, Germany; 2 Data.Org, New York, NY USA; 3 London School of Hygiene & Tropical Medicine, London, UK; 4 EcoHealth Alliance, New York, NY USA; 5 Public Health Agency of Canada, Ottawa, Canada

**Keywords:** Pandemic intelligence, Epidemic intelligence, Cross-community collaboration, Co-creation, Artificial intelligence, Public health, Communities of practice

## Abstract

The twelfth session of the WHO Pandemic and Epidemic Intelligence Innovation Forum focused on how cross-community collaboration can accelerate progress in pandemic and epidemic intelligence. The session explored how the Collaboratory, a WHO digital initiative connecting diverse communities of practice, fosters co-creation, supports the development of collaborative analytical tools, and strengthens the translation of evidence-base into public health action. Through a combination of short presentations and a panel discussion, the session examined key Collaboratory initiatives, including the Global Repository of Epidemiological Parameters (grEPI), AI-enabled pipelines for evidence synthesis, the Epiverse toolkit for outbreak analysis, and semantic search tools to improve the discoverability of epidemiological resources. Discussions addressed challenges of integration into national public health systems, governance models, sustainability, capacity-building, AI reliability, and ethical use. Across the session a common message emerged: advancing epidemic intelligence requires not only new tools but also sustained collective action, trust, and shared ownership across sectors, disciplines, and regions.

## Introduction: advancing epidemic intelligence through collective action

As the volume, complexity, and urgency of data for pandemic and epidemic intelligence continue to grow, the need to connect people across disciplines has become increasingly critical. Strengthening collaboration between analytics experts, operational teams, decision-makers, and technology developers is essential to ensuring that data, tools, and insights are developed together and co-owned, and that they translate into actionable public health decision-making.

The twelfth session of the WHO Pandemic and Epidemic Intelligence Innovation Forum held on March 24th 2025 brought together experts from the WHO Hub for Pandemic and Epidemic Intelligence [1], Ecohealth Alliance [2], the London School of Hygiene & Tropical Medicine [3], the Public Health Agency of Canada [4], and data.org [5] to discuss how cross-community collaboration can accelerate progress in pandemic and epidemic intelligence. Through a combination of short presentations and a panel discussion, the session examined key initiatives of the WHO Collaboratory, a curated digital environment and global community, designed to connect diverse groups, share knowledge, co-create analytical tools, and collaborate on strengthening pandemic and epidemic intelligence.

Participants reflected on integration challenges, governance models, capacity-building needs, and the future priorities for collective action. Across all discussions, a shared message emerged: that sustainable, inclusive, and co-created approaches are fundamental to advancing the global epidemic intelligence ecosystem.

## The Collaboratory: enabling co-creation and cross-community collaboration

The Collaboratory is a WHO initiative that serves as both a digital platform and a global convening mechanism, designed to break down silos between sectors, disciplines, and regions in the field of pandemic and epidemic intelligence [6]. Hosting more than seventeen active communities of practice (CoPs), the Collaboratory provides a structured space where public health agencies, academic researchers, technology developers, non-governmental organizations, and international institutions can work together to address shared challenges. It aims at establishing a digital environment where the pandemic and epidemic intelligence community can come together to address critical challenges that affect the way data is accessed, analyzed, visualized, and communicated for better pandemic and epidemic policy and response decision making. Emphasizing a culture of collective effort, the Collaboratory seeks to reconceptualize incentives for pooling resources and capacities. In doing so, it aspires to promote collaborative problem-solving and best practice formulation.

The core goal of the Collaboratory is to foster co-creation where stakeholders collaborate in shaping the design of global public health tools, their implementation, and adaptation from the outset. By doing so, the Collaboratory aims to bridge two persistent gaps in the global health landscape. On one hand, operational teams working in national or local public health settings often possess rich, context-specific data but have limited in-house capacity for advanced analytics. On the other hand, analytics communities and technology developers may bring cutting-edge methods but lack sufficient understanding of the day-to-day realities, constraints, and needs faced by practitioners and decision-makers.

By providing a shared space for dialogue and work, the Collaboratory enables these groups to connect, ensuring that analytical innovations are not developed in isolation but shaped by continuous feedback from the communities they are meant to serve. For example, the dengue analytics community of practice brings together surveillance experts, climate scientists, modelers, and public health officials to co-develop approaches for forecasting dengue outbreaks, ensuring that models are not only scientifically rigorous but also usable and meaningful for those managing health responses on the ground.

The platform also hosts communities focused on mpox analytics, influenza surveillance, epidemic intelligence from open sources, and the development of outbreak response tools for current and future threats, among others. Across these CoPs, the Collaboratory supports a diverse range of activities, including joint data analysis, sharing of case studies, identification of research gaps, and dissemination of best practices.

The Collaboratory is not just a digital environment; it is also a social infrastructure designed to build relationships, trust, and a shared sense of purpose among its members. By bringing together actors from across countries, sectors, and technical backgrounds, it helps create a global network of peers who can learn from each other, amplify local innovations, and jointly advance the state of epidemic and pandemic intelligence.

## Collaborative innovation integrated into public health systems

A good example of the Collaboratory’s integrative function is the Global Repository of Epidemiological Parameters (grEPI), developed through collaboration between WHO, Imperial College London, the London School of Hygiene & Tropical Medicine, and the Public Health Agency of Canada. grEPI provides an open-access, centralized source for key epidemiological parameters like reproduction numbers, incubation periods, and serial intervals, that are essential for supporting timely and accurate disease modellings and risk assessments. What makes grEPI distinctive is not just the technical architecture but also the fact that it is shaped by the inputs and needs of a diverse group of modelers, epidemiologists, public health practitioners, and policymakers across geographies.

Another critical initiative within the Collaboratory ecosystem is the AI-enabled evidence synthesis pipeline, led by the Public Health Agency of Canada in collaboration and WHO [7]. While grEPI focuses on curating and centralizing validated parameters, the AI pipeline addresses the upstream challenge of systematically gathering, reviewing, and synthesizing the research evidence that informs those parameters. By combining artificial intelligence (AI) with human oversight, the pipeline accelerates the production of systematic reviews and meta-analyses, ensuring that the data feeding into grEPI and other decision-making tools is timely, rigorously assessed, and relevant [8, 9].

The Epiverse toolkit, co-developed by the London School of Hygiene & Tropical Medicine (LSHTM) and other organizations through Data.org Epiverse-TRACE initiative, is another example of a collaboration aimed at supporting the generation of public health evidence-base, offering a modular suite of R packages designed for data cleaning, outbreak modelling, and intervention assessment, supported by open-source licensing and open development, training materials, and user guides that make advanced analytics accessible to experts and non-experts across varied settings [10].

Complementing this, Data.org also presented its semantic search and mapping tool, that uses an AI-driven system to organize over 130 epidemiological R packages into a searchable, thematic space [11]. This tool empowers practitioners to identify relevant R packages even without deep technical knowledge, reducing duplication and fostering a more collaborative global ecosystem for data analysis.

## Building sustainable, scalable, and accessible collaborative solutions

During the panel discussion, the question of sustainability and scalability was explored in depth, with panelists reflecting on what it takes to ensure that these innovations are not only created but maintained, updated, and embedded over time. They emphasized that technological innovation must be accompanied by community stewardship, governance, and accessibility to achieve lasting impact [12, 13].

Several key points emerged. First, sustainability is not solely a matter of financial resources but is strongly tied to building networks of communities where no institution becomes a sole critical dependency [14]. The Epiverse initiative, for example, intentionally connects to multiple partner institutions, drawing on diverse streams of expertise and funding to avoid dependence on any one actor.

Second, sustainability depends on designing tools that generate routine, practical value for users [15]. Panelists agreed that when tools demonstrably save time, simplify workflows, or improve decisions, they are far more likely to be adopted and maintained. The AI-enabled evidence synthesis pipeline, when paired with grEPI, exemplifies this point: by automating labor-intensive evidence reviews and feeding directly into parameter repositories used in real-world modelling, the system ensures that outputs are not merely theoretical but operationally useful.

Finally, the panelists stressed that sustainability includes building capacity and ownership at local and national levels [16]. Rather than delivering one-size-fits-all solutions, the Collaboratory initiatives are sustainable when they enable local adaptation, training, and co-development. Whether through modular software design, human-in-the-loop AI, or flexible governance models, sustainability is ultimately a reflection of the Collaboratory’s commitment to collective stewardship and community-driven innovation.

## Ensuring reliable, ethical, and environmentally responsible AIAI innovation

Beyond questions of institutional sustainability, the integration of AI across the Collaboratory initiatives raises important challenges of environmental impact, reliability, transparency, and accountability. This was addressed directly in the panel discussion, where speakers acknowledged that while AI can accelerate processes such as evidence synthesis, semantic search, and outbreak forecasting, it must be applied carefully, with rigorous validation, contextual adaptation, and human oversight [17].

In this context, the Public Health Agency of Canada highlighted the importance of using minimal, purpose-driven modelling approaches wherever possible to reduce unnecessary energy consumption and ensure that innovations align with environmental sustainability goals [18]. Panelists also discussed the value of Agentic AI approaches, where the system can assess its own confidence and pass decisions to human reviewers when thresholds are not met to balance automation with expert judgment. Such human-in-the-loop designs ensure that experts can review, interpret, and adapt AI outputs for local use, avoiding “black box” risks and reinforcing trust in the system [19].

Panelists argued that the reliability of AI is not just a technical matter but part of a broader ecosystem of ethical governance, accountability, and user engagement. They emphasized that for AI innovations to properly serve the needs of public health systems, they must be transparent, auditable, and designed in partnership with the communities that will use them [20, 21].

## Embedding innovation into national public health workflows and decision-making

During the panel discussion, participants were asked how they see such analytical innovations being integrated into national public health agency workflows and supporting decision-making. Many national agencies face increasing external pressure, from media, political actors, or the public, to provide rapid analytic insights during emergencies.

During an emergency, the evolving evidence landscape makes it even more critical for the implementation of tools like grEPI and the AI pipeline, but those need to be designed to complement, rather than disrupt, existing workflows, offering flexible points of entry, clear interfaces, and robust human support [22, 23]. For example, in some settings, public health teams may rely on modular components or packaged outputs; in others, they may want direct access to databases or analytic code. Together, these initiatives can work toward optimizing the evidence-to-action loop, allowing public health agencies to move from data to insight to intervention more efficiently and confidently. Their integration can support evidence generation and streamline workflows in outbreak response making it easier for national agencies to rapidly access validated parameters, update modelling inputs, and inform public health measures in near real time.

In this context, panelists emphasized that integration is not simply a technical challenge but an institutional one: it requires trust between national agencies and the collaborative initiatives offering tools and platforms. They noted that agencies are more likely to adopt external innovations when they are involved early in co-creation, when tools are transparent and well-documented, and when outputs can be easily tailored to local policy questions [24–26]. It is not just about technological fit but about capacity-building and relationship-building. To effectively support national decision-making, The Collaboratory projects provide training, documentation, and ongoing engagement, ensuring that local teams understand how to use tools, adapt them to their needs, and interpret outputs in ways that align with their operational and policy contexts. This relational and capacity-focused approach, panelists argued, is essential for moving from innovation as proof of concept to innovation as embedded practice in public health systems.

## Strengthening the Collaboratory as a community of trust, openness, and experimentation: the way forward

The Collaboratory is evolving beyond being just a digital environment; it is becoming a dynamic global community where trust, openness, and experimentation are intentionally cultivated and nurtured. This transformation was vividly reflected at the 2025 Collaboratory Summit, which took place in Berlin days before the Innovation Forum and brought together eighty-five participants across four days of interactive workshops centered on governance, culture, and innovation. Both during the Summit and the Forum session, participants stressed that governance is not a background administrative detail but a central determinant of whether collaborative innovation for public health succeeds or falters.

Good governance must include clear roles, transparent processes, fair and inclusive decision-making, and, critically, the flexibility to adapt as challenges and opportunities evolve. Building shared norms and values, creating spaces for inclusive dialogue, and embedding participatory governance into daily operations is essential to making the Collaboratory a trusted, community-owned space. For example, the launch of the Innovation Forum community space on the Collaboratory marks a meaningful step forward in this direction, offering a digital venue where members can exchange lessons about a very diverse set of topics, propose new initiatives, and co-shape the future of the Forum itself.

As the session concluded, participants agreed on several key priorities for the future of collaborative pandemic and epidemic intelligence. There was broad consensus that strengthening co-creation at every stage of the innovation process is essential, ensuring that local perspectives, needs, and expertise are embedded into global tools and approaches. Sustaining collaboration will require continuous investment in governance models that balance openness with accountability and distribute leadership across the network.

Importantly, that success will depend on expanding capacity-building efforts that support both well-resourced and under-resourced public health teams, enabling more actors to engage meaningfully with tools, data, and collaborative projects. The Collaboratory is positioned to play a catalytic role in connecting and amplifying these efforts, providing not only the technical infrastructure but also the virtual social space where diverse actors can come together to test ideas, share learning, and build the collective intelligence needed to meet the epidemic challenges of today and tomorrow.

## Abbreviations

AI: Artificial intelligence
CoP: Community of practice
grEPI: Global Repository of Epidemiological Parameters
LSHTM: London School of Hygiene & Tropical Medicine
WHO: World Health Organization
